# Case Report: Tumor Lysis Syndrome After Quad Shot in Diffuse Large B-Cell Lymphoma

**DOI:** 10.1016/j.adro.2023.101265

**Published:** 2023-05-05

**Authors:** Patrick Newbury, Ritvik Ganguly, Christina Henson

**Affiliations:** a Department of Radiation Oncology, University of Oklahoma Health Sciences Center, Oklahoma City, Oklahoma; b University of Oklahoma College of Medicine, Norman, Oklahoma

## Introduction

Tumor lysis syndrome (TLS) is a metabolic condition often resulting from the initiation of cancer therapy and is commonly associated with hematologic malignancies (HMs). TLS is due to the rapid lysis of cancer cells rich in purines, phosphate, and other intracellular components, leading to numerous systemic effects. These include hyperuricemia, hyperphosphatemia, hypocalcemia, cardiac arrythmia, seizures, renal dysfunction, and even death.^1^ While TLS is more commonly caused by cytotoxic systemic therapy, there are reports of radiation-induced as well as spontaneous cases arising due to high-grade, highly proliferative, and bulky malignancies such as non-Hodgkin lymphomas.^2^

The incidence of TLS varies significantly among the different HMs, patient populations, and therapy regimens. A multicenter study including adults and children having acute leukemia or non-Hodgkin lymphomas reported overall laboratory and clinical TLS incidence to be 18.9% and 5.0%, respectively.^3^ A retrospective analysis of the Henry Ford health system found an overall laboratory TLS incidence rate of 9.3% and clinical TLS rate of 6.7%.^1^ Thus, clinically significant TLS is an uncommon occurrence in most radiation therapy practices, which may contribute to some groups being ill prepared to handle this oncologic emergency.

While TLS management involves aggressive hydration and managing uric acid, severe renal impairment may occur despite best practices.^1^ Prevention and preparedness while delivering cancer treatments are the most effective strategy against TLS. Compared with cytotoxic modalities, radiation therapy as the primary cause of TLS has been less commonly described in the literature.^4^ We report a case of TLS that occurred after the first dose of a quad shot in a patient with diffuse large B-cell lymphoma (DLBCL).

## Case and Clinical History

### Chronology of previous cancer treatment

We present the case of a 26-year-old female patient with a diagnosis of stage III chronic lymphocytic leukemia with trisomy 12. She achieved remission after 6 cycles of fludarabine, cyclophosphamide, and rituximab. A few years later, she relapsed and experienced significant cervical lymphadenopathy, fatigue, weakness, and weight loss. Six years after diagnosis, an excisional lymph node biopsy was performed on the right axilla again revealing chronic lymphocytic leukemia.

The patient was started on a bendamustine and rituximab protocol. Unfortunately, biopsy of a left axillary lymph node revealed transformation to DLBCL consistent with Richter syndrome (Ki-67 was 70%-80%, PAX5 positive, and cyclin D1 negative). Three months later, a positron emission tomography scan revealed Ann Arbor stage III, Deauville class 4 disease. She was started on cyclophosphamide, doxorubicin, prednisone, rituximab, and vincristine (R-CHOP), which was later modified to rituximab, gemcitabine, and oxaliplatin (R-GemOx), and then rituximab, ifosfamide, carboplatin, and etoposide (RICE) as a bridge to chimeric antigen receptor T-cell therapy. After 6 cycles, a positron emission tomography scan again showed Deauville 4 disease with some lesions improved, but some had progressed and were causing neck soreness and decreased range of motion. It was at this point that, due to the refractory nature of her disease and how quickly it tended to progress off therapy, she was referred to us for a short course of temporizing radiation while awaiting platelet recovery before chimeric antigen receptor T-cell therapy.

### TLS occurrence with quad shot

**Figure 1:**
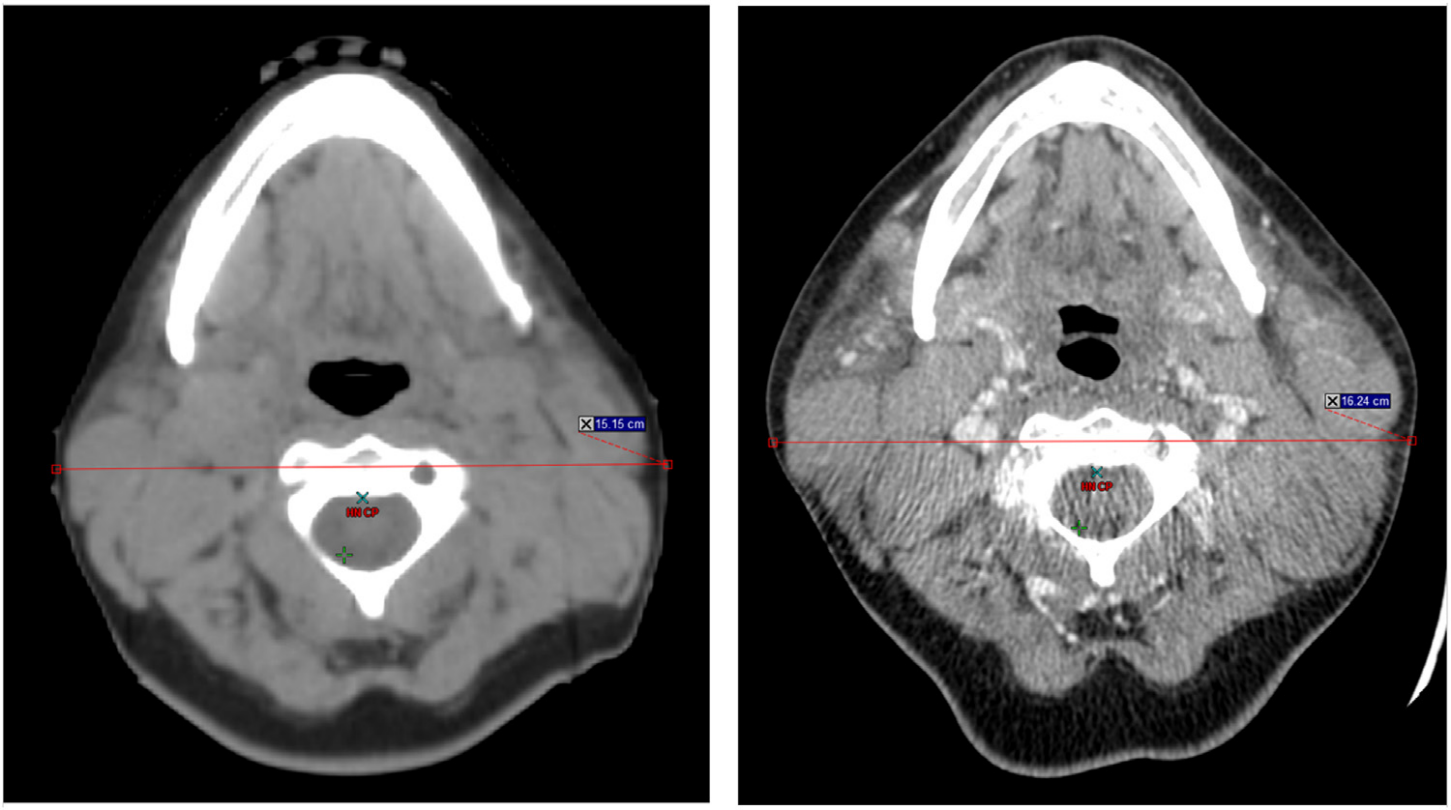
Computed tomography scan of neck before radiation therapy (left) and after radiation therapy (right). Before, neck diameter was 15.15 cm. After, it was 16.24 cm with marked change in the shape of soft tissues.

**Figure 2:**
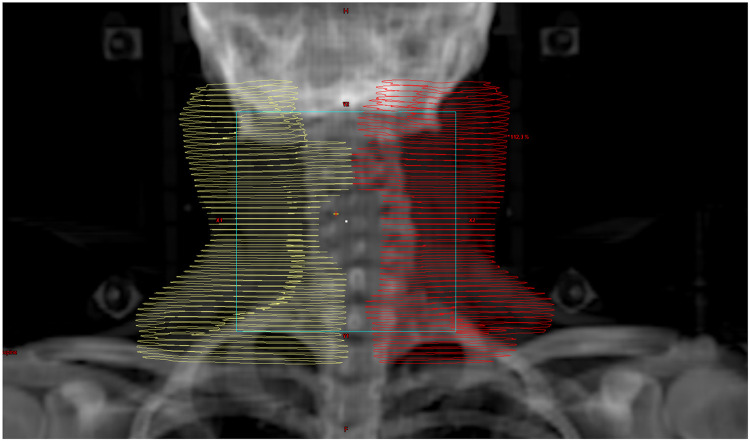
Anterior/posterior digitally reconstructed radiograph demonstrating large bilateral cervical planning target volumes.

The patient received the first fraction of a quad shot, with 14 Gy to be administered in 4 fractions over 2 days. This was to be delivered to her largest and most poorly responding nodes, which included bilateral cervical chains and left axillary volumes. The total volume irradiated was 1190.85 cc, with over 900 cc being represented by cervical nodes as demonstrated in Figure 1. When she came back for her second fraction in the late afternoon, she was experiencing marked fever, malaise, dry mouth, tachycardia as high as 160 beats per minute, and a temperature of 100.6°F. She reported a headache. Her neck was also markedly more swollen on the left, but all lesions treated with radiation in the morning were swollen (see Fig 2).

After monitoring her for an hour, she mildly improved. The second fraction was delivered, and she was admitted to the hospital for further observation. Upon admission, her vitals were abnormal as demonstrated in Table 1.

**Table 1:** Relevant vital signs

Day and time	Temperature (°C)	Pulse (bpm)	Respirations	BP (mmHg)	Pulse ox (%)
Day before RT	36.5	88	16	118/96	96
2 PM day of RT	38.1	160	17	114/79	98
6 PM day of RT	38.9	139	16	109/77	99
9 PM day of RT	37.1	108	18	94/60	96

*Abbreviations:* BP = blood pressure; bpm = beats per minute; ox = oxygen; RT = radiation therapy.

As we failed to alert the primary team to the likely diagnosis of TLS, the hospitalist team started the patient on broad spectrum antibiotics and intravenous dexamethasone 4 mg every 6 hours in the context of recent 40 mg every day for the previous 2 days for thrombocytopenia. Her symptoms improved overnight, her vitals normalized, and she completed her final 2 fractions the next day without further incident. Relevant laboratory results are shown in Table 2.

**Table 2:** Chemistry panel before and during symptoms of tumor lysis syndrome

Relevant chemistry panel dates	Day of RT	Next day
Time	18:45	03:50
Sodium (136-145 mEq/L)	139	142
Potassium (3.5-5.1 mEq/L)	3.9	4.0
Chloride (97-109 mEq/L)	105	107
Carbon dioxide (23-32 mEq/L)	26	29
Anion gap (4-14)	8	6
BUN (7-17 mg/dL)	13	10
Creatinine (0.7-1.1 mg/dL)	0.49	0.57
Glucose (66-111 mg/dL)	110	141
Uric acid (2.6-6.0 mg/dL)	5.5	–
Calcium (8.7-10.1 mg/dL)	9.1	9.4
Phosphorus (2.5-4.5 mg/dL)	3.3	4.7
Magnesium (1.6-2.6 mg/dL)	1.6	1.9
Total alk phosphatase (63-157 U/L)	121	107
Lactate dehydrogenase (112-236 U/L)	414	–
High-sensitivity C-reactive protein (mg/L)	2.9	–

*Abbreviations:* alk = alkaline; BUN = blood urea nitrogen; RT = radiation therapy.

## Discussion

TLS is an uncommon but possibly deadly metabolic cascade that can occur spontaneously, due to cytotoxic therapies or from radiation therapy. It is characterized by several metabolic changes. These include elevation of uric acid, hypocalcemia, hyperphosphatemia, hyperkalemia, and renal failure.^5^ There are risk factors for TLS such as high tumor cell proliferation rate, high sensitivity to therapy, large tumor burden, bulky disease, impaired renal function, pretreatment hyperuricemia/hyperphosphatemia, renal disease, oliguria, and dehydration. These factors should be considered before providing therapy, whether systemic or radiation.^6^ An expert consensus published guidelines to identify both patients and treatments at risk, and DLBCL is in the highest risk group.^7^ However, our practice did not have a TLS risk assessment as part of our workflow, and this led to suboptimal care. If the patient's TLS was more severe due to intrinsic tumor characteristics, or if we had covered additional nodal volumes, this may have been fatal.

There are a few reported cases of radiation-induced TLS, with doses as low as 1.5 Gy to HMs and 3 Gy to solid histologies not known for their high risk of TLS.^8^, ^9^, ^10^, ^11^, ^12^ While additional individual risk factors certainly contributed to TLS in those cases, we also wonder if a lower-dose quad shot might have been indicated given the radiosensitive nature of DLBCL. There are current efforts to dose deescalate in orbital mucosa-associated lymphoid tissue,^13^ gastric mucosa-associated lymphoid tissue,^14^ and follicular lymphoma.^15^ It stands to reason that the dose required to palliate this DLBCL would not be the same as a similar-size squamous cell carcinoma, for which the regimen was developed.^16^ However, precisely how to modify the regimen in this context has not yet been explored.

There were numerous uncommon changes observed after radiation therapy in our patient. Providers were unable to rapidly diagnose TLS, which led to suboptimal care. Using the Cairo-Bishop criteria for TLS (Table 3),^17^ her only salient change was hyperphosphatemia. While this does not fulfill the laboratory criteria, a clinical diagnosis of TLS was made due to cardiac arrhythmia. In addition, the significantly elevated, though not double the upper limit of normal, pretreatment LDH may have been a hint at greater susceptibility to TLS.

**Table 3:** Tumor lysis syndrome criteria set forth by Cairo and Bishop^17^

**Figure 3:**
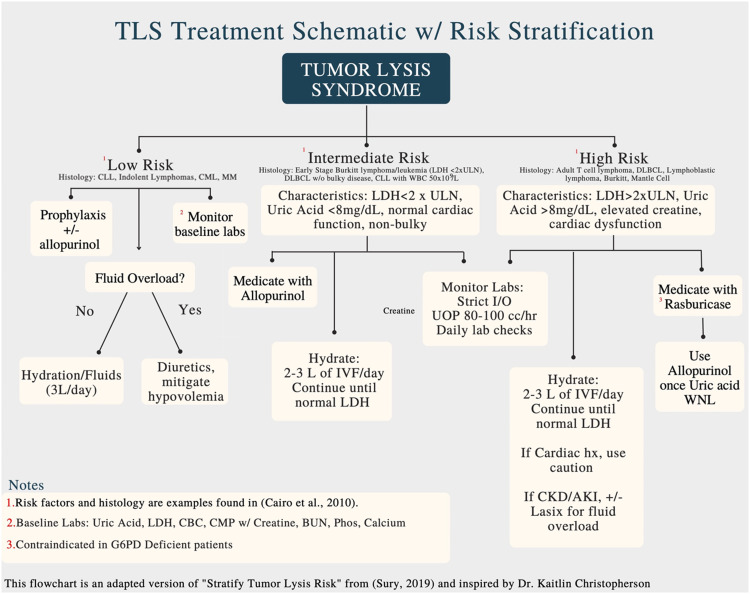
Tumor lysis syndrome (TLS) risk stratification and treatment algorithm. *Abbreviations:* AKI = acute kidney injury; BUN = blood urea nitrogen; CBC = complete blood count; CKD = chronic kidney disease; CML = chronic myelogenous leukemia; CLL = chronic lymphocytic leukemia; CMP = comprehensive metabolic panel; DLBCL = diffuse large B-cell lymphoma; IVF = intravenous fluids; LDH = lactate dehydrogenase; MM = multiple myeloma; ULN = upper limit of normal; UOP = urine output; WNL = within normal limits.

For patients experiencing acute TLS, there are several important biomarkers to monitor, including whole blood count, uric acid, phosphate, and calcium. The TLS classification system before treatment aids with risk stratification. Given that DLBCL is a high-risk entity, prophylaxis with allopurinol or rasburicase may be indicated.^5^ Prophylaxis for high-risk patients is supported by other case reports.^11^ The important biomarkers for TLS should be monitored at least daily. Refer to Figure 3 for risk-assessment and treatment algorithm. Our case serves as a reminder for practices which see very few histologies at high risk for TLS to remain vigilant for this syndrome. This case also raises the question as to what radiation dose is appropriate in these patients and if TLS is a dose-limiting/dependent entity.

## Conclusion

TLS is an uncommon side effect of cytotoxic cancer treatment, and it is sometimes associated with radiation therapy in the literature. To our knowledge, this is the first case report presenting TLS in a patient with DLBCL who underwent palliative quad shot. This case also highlights the importance of recognizing patients at high risk for TLS, recognition of the syndrome, and communication within a treatment team.

## Disclosures

The authors declare that they have no known competing financial interests or personal relationships that could have appeared to influence the work reported in this paper.
